# Wharton's Jelly Mesenchymal Stem Cell Administration Improves Quality of Life and Self-Sufficiency in Children with Cerebral Palsy: Results from a Retrospective Study

**DOI:** 10.1155/2019/7402151

**Published:** 2019-05-02

**Authors:** Dariusz Boruczkowski, Izabela Zdolińska-Malinowska

**Affiliations:** Polski Bank Komórek Macierzystych S.A./FamiCord Group (Polish Stem Cell Bank), Jana Pawła II 29, Warsaw, Poland

## Abstract

The aim of this paper was to describe the outcome of the therapeutic administration of allogenic mesenchymal stem cells obtained from Wharton's jelly (WJ-MSCs) in children with cerebral palsy (CP) during a medical therapeutic experiment. We retrospectively analyzed the records of 109 patients recruited in daily clinical practice. Each patient received 1–10 injections and was examined by the same neurologist (study investigator (SI)) on the day of each infusion. The SI used a 6-point Likert scale to assess the quality of life (QoL) and self-sufficiency of the patients on the basis of the neurological examination. Children with >50% follow-ups after this administration were included into the quantitative analysis. In addition, the assessments of the parents and other health care professionals were obtained for 23 patients and compared with those of the SI. Forty-eight of 54 analyzed patients (88.9%) achieved some improvement in health status. Forty-eight (88.9%) patients experienced an increase in their QoL, and 21 patients (38.9%) achieved an increase in their self-sufficiency level. Improvement was achieved in 17 areas. Adverse events were mild and temporary except one case of epilepsy deterioration leading to treatment discontinuation. Age, body mass, and cell dose were not significant predictors of QoL response, contrary to epilepsy; developmental breakthrough was dose-dependent.

## 1. Introduction

Cerebral palsy (CP) is the most common motor disorder in children [1], decreasing their quality of life (QoL) and level of self-sufficiency (SS). A meta-analysis of 30 out of 1366 papers describing this disability revealed that 3 in 4 CP patients were in pain, 1 in 2 had an intellectual disability, 1 in 3 could not walk, 1 in 3 had hip displacement, and 1 in 4 could not talk. In addition, 1 in 4 CP patients had epilepsy, 1 in 4 had a behaviour disorder, 1 in 4 had bladder control problems, 1 in 5 had a sleep disorder, 1 in 5 dribbled, 1 in 10 was blind, 1 in 15 was tube-fed, and 1 in 25 was deaf [2]. Because there is no single cause of CP, the efficacy of care and rehabilitation strategies is limited, and high expectations are placed on cell-based therapies, especially by the patients' parents [3–5].

The first therapeutic administration of stem cells for CP happened in 2009. The recipient was a 2.5-year-old boy with CP caused by global hypoxic-ischemic brain damage due to cardiac arrest [6]. After autologous umbilical cord blood (UCB) administration, the patient's motor control, spastic paresis, social contact (smile), and speaking skills improved. After that, the high efficacy and safety of UCB and MSCs have been described in several case reports [7–12]. In these papers, improvement was noted using functional scales, in motor functions, through electroencephalography (EEG); in bladder/bowel control; and in communication skills, with no serious adverse events (AEs).

In Poland, doctors may engage in two kinds of medical studies: therapeutic experiments and research experiments (clinical studies) (*Journal of Laws of 2008*, No. 136, item 857). While the main goal of a research experiment is to expand medical knowledge, the aim of a therapeutic experiment is to improve the patient's health through the use of new—or only partially tested—diagnostic, therapeutic, or prophylactic methods. A therapeutic experiment can be performed when the current treatment options for a disease are ineffective or insufficient. Sometimes, participation in a therapeutic experiment is a patient's only possibility of treatment. Although therapeutic experiments are not so strictly regulated as clinical studies and therefore cannot be matched in terms of data quality, they still possess a scientific value. The aim of our paper was to retrospectively analyze the efficacy and safety of WJ-MSC administration in children with CP participating in a therapeutic experiment.

## 2. Materials and Methods

### 2.1. Product Description

The WJ-MSCs were donated by healthy Polish newborns born naturally or via cesarean section. Maternal health was verified using a medical questionnaire, after which parents signed an informed consent form. The approval of the appropriate Bioethics Committee was obtained before tissue collection.

After harvesting, the umbilical cords (UC) were transported to the laboratory under monitored conditions and processed within 48 h of delivery. First, UC were disinfected by washing in a sterile saline solution supplemented with an antibiotic-antimycotic mixture (Gibco) and were then dissected and stripped of any blood vessels. Using a sterile lancet, Wharton's jelly was minced into 2 cm^3^ pieces, which were placed into 6-well plates covered with MSC Attachment Solution (Biological Industries) according to the manufacturer's instructions. The tissue explants were cultured in NutriStem® XF serum-free medium (Biological Industries) supplemented with NutriStem®XF Supplement Mix (Biological Industries) and an antibiotic-antimycotic solution (Gibco) and incubated at 37°C in 5% CO_2_ in air. After 1-2 hours, the nonadherent cells were washed off, and the attached cells were further expanded. The tissue explants were removed after 2-3 weeks of culture. When the adherent cells reached 90% confluence, they were passaged and reseeded for further expansion at 1.2 × 10^4^ cells/cm^2^ in a 75 cm^2^ tissue culture flask (BD). To evaluate their numbers, the cells were detached using a trypsin solution (Biological Industries) and counted in a haemocytometer. When a sufficient number of cells was reached, they were transferred to a freezing bag for cryopreservation. The cells were resuspended in a 10% (*v*/*v*) DMSO (WAK-Chemie) solution in human albumin (CSL Behring), frozen in a controlled-rate freezer (Sy-Lab IceCube 14S), and stored in the vapour phase of liquid nitrogen.

UC-derived MSCs were characterized by immunophenotyping according to the criteria for defining MSCs described by Dominici et al. [10]. Briefly, upon reaching 60-80% confluence, cells were trypsinized and incubated in the dark for 30 min with fluorochrome-conjugated antibodies against MSC-negative (CD34 FITC, CD14 FITC, CD19 FITC, CD 45 FITC, and HLA-DR FITC) and MSC-positive (CD 73 PE, CD90 PE, CD105 PE, and HLA ABC FITC) surface markers. Mouse anti-IgG1 FITC and anti-IgG1 PE were used as controls. The cells were then washed with a washing solution, resuspended in Cell Fix solution, and analyzed using a BD FACSCalibur cytometer.

The postthaw MSC viability was determined based on that of a thawed reference sample. The cells were stained with trypan blue, and live cells were counted in a haemocytometer. Neither the proliferation rate nor any other indicators of cellular senescence were monitored. However, only 7 patients received cells after the fifth passage; all other patients received cells that were at passage 4 or lower.

Good manufacturing practice was followed throughout the process. The final medicinal product complied with the Chief Pharmaceutical Inspectorate's requirements in terms of the unit volume; number, vitality, and morphology of the cells; microbiological purity; results of serological tests; absence of endotoxins; and immunophenotype of the cells. Before administration, the cells were placed in a water bath and thawed at 37°C.

### 2.2. Patients

We performed a retrospective analysis of the medical records of Polish patients with CP who received MSC infusions as part of a therapeutic experiment between 2014 and 23 June 2018. All the data were fully anonymised before we accessed them. Before the beginning of the study, approval was obtained from the Bioethics Committee at the Medical University of Lublin, Lublin, Poland (KE-0254/274/2014, KE-0254/240/2015, and KE-0254/242/2016); the Bioethics Committee at the Regional Chamber of Physicians and Dentists in Lublin, Poland (152/2018/KB/VII); and the Bioethics Committee at Andrzej Frycz Modrzewski Cracow University, Cracow, Poland (24/2016). The study was conducted in accordance with the Declaration of Helsinki. An informed consent form, including consent for the use of the patient's medical data for statistical analysis, was signed by the patients' parents before the MSC administration. All patients were informed about the possibility of publication before the form was signed.

The patients were recruited by a neurologist in daily practice. Treatment consisted of 1-5 intravenous stem cell injections per treatment course. Up to two treatment courses were permitted. Four standard stem cell doses of 10, 20, 30, or 40 × 10^6^ MSCs/injection were used. The total stem cell count received varied according to the weight of the patient, but the approximate dose per kilogram was 1 × 10^6^ MSCs. Injections were administered every two months, after patient qualification by the study investigator (SI).

### 2.3. Clinical Assessment

All the patients were examined by the same SI, a neurologist, on the day of each infusion. The examinations consisted of a neurologic and paediatric qualitative assessment. The quality of life (QoL) and self-care level of the patients were evaluated based on a physical examination and a medical interview. The results were described using a 6-point Likert scale, in which 0 corresponded to a lack of improvement, and 5 corresponded to an excellent improvement. Scores for QoL and SS level were obtained for each examination and compared to the results of the previous examination. Spontaneous reports by the patients' parents and third-party therapists such as physiotherapists, sensory integration specialists, and pedagogues were obtained from the parents by the Polish Stem Cell Bank and included in this retrospective analysis.

As mentioned in the previous section, four standard stem cell counts per injection were used, and the body mass of the patients fluctuated throughout the study. Hence, the real stem cell dose range was calculated using the lowest and highest body masses recorded during therapy, and the highest and lowest stem cell doses used per injection.

### 2.4. Data Processing and Statistical Analysis

Qualitative data were coded as improvement, deterioration, or no change. The following areas were evaluated: muscle tension, muscle strength, gross motor development, fine motor development, nutritional functions, senses, excretion/defecation control, sleeping, circulation, epilepsy attacks, drug dosage, emotions, communication, attention, cognitive functions, engagement (motivation/initiative/cooperation), and social interactions. These results are presented as a number and percentage, calculated using Microsoft Excel. In addition, the nonparametric sign test was performed using the statistical software Statistica 13.0. Statistical significance was considered at *p* ≤ 0.05.

The averages for QoL and self-sufficiency were calculated as the sum of individual values divided by number of assessments. The difference between the median QoL score and the median self-service score per administration was assessed using a Kruskal-Wallis rank test with a Bonferroni correction. Subsequently, the results were categorized using a binary system based on the number of past administrations (1 vs. >1, ≤2 vs. >2, and ≤3 vs. >3) and compared using the Mann-Whitney *U* test.

The minimum single WJ-MSC dose was calculated as the minimum cell count administered in one injection divided by the maximum body mass during one treatment course. The maximum single WJ-MSC dose was calculated as the maximum cell count administered in one injection divided by the minimum body mass during one treatment course. The minimum total WJ-MSC dose per kilogram of body mass was calculated by multiplying the minimum cell count administered in one injection by the number of administrations and dividing by the maximum body mass during the whole treatment course. The maximum total WJ-MSC dose per kilogram of body mass was calculated by multiplying the maximum cell count administered in one injection by the number of administrations and dividing by the minimum body mass during one treatment course.

The age, minimum and maximum single and total doses, and average QoL improvement per administration followed nonnormal distributions. Therefore, Kendall's tau and Spearman *R* coefficients were used to calculate the nonparametric correlation between average improvement in QoL per past administration and age, single dose per kg of body mass, and the minimum total stem cell dose. The nonparametric correlation between QoL after the first cell administration (early response) and average QoL in the following administrations was assessed with the same method. We also calculated these correlations for the average improvement in QoL per collected assessment to minimize the impact of missed follow-ups. After binary categorization, the results were verified using Yates' chi-square test.

## 3. Results

### 3.1. Patients

We analyzed the data of 107 children aged 17–204 months (17 years) (median age: 61 months, interquartile range (IQR) 39–117 months) at baseline. The minimum body mass during the course of therapy ranged from 7.0 to 75.0 kg (median: 15.0 kg, IQR 11.3–20.0 kg). The maximum body mass ranged from 7.0 to 86.0 kg (median: 16.5 kg, IQR 12.6–23.0 kg). The difference in body mass at the beginning and the end of therapy ranged from 0 to 21 kg (median: 0.7 kg, IQR 0–2 kg). The minimum single WJ-MSC dose ranged from 0.5 × 10^6^ to 1.6 × 10^6^ WJ-MSCs/kg (median 0.9 × 10^6^ WJ-MSCs/kg, IQR 0.77 × 10^6^–1.05 × 10^6^ WJ-MSCs/kg). The maximum single WJ-MSC dose ranged from 0.5 × 10^6^ to 2.14 × 10^6^ WJ-MSCs/kg (median 1.0 × 10^6^ WJ-MSCs/kg, IQR 0.87 × 10^6^–1.25 × 10^6^ WJ-MSCs/kg). The minimum total WJ-MSC dose per kilogram of body mass ranged from 0.7 × 10^6^ to 11.5 × 10^6^ (median 3.97 × 10^6^, IQR 3.0 × 10^6^–5.0 × 10^6^). The maximum total WJ-MSC dose per kilogram of body mass ranged from 0.71 × 10^6^ to 13.6 × 10^6^ (median 4.4 × 10^6^, IQR 3.3 × 10^6^–5.7 × 10^6^). Thirty-one (28.4%) children had epilepsy.

### 3.2. Sample Size

Follow-ups were available for 90 (84.1%) children, but many reports were incomplete. All collected follow-ups were used to assess the safety of the therapy. At least 50% of expected follow-ups were available for 54 children, including 59% of those who received at least two infusions and therefore had at least one assessment. This subpopulation was used to calculate the average improvement in QoL and self-service as well as to assess the number of significantly improved areas with the sign test. Parental assessment and additional documentation were supplied by the parents of 23 children and were used to evaluate treatment efficacy (Table 1).

The subgroup included in the QoL and SS calculations had similar characteristics to the whole study population. The age of the patients was 17–201 months (16 years and 9 months) at baseline (median age: 62 months, interquartile range (IQR) 37–123 months). The minimum body mass during therapy ranged from 7.5 to 75.0 kg (median: 15 kg, IQR 11.0–22.75 kg). The maximum body mass ranged from 10.0 to 86.0 kg (median: 16.25 kg, IQR 12.0–25.5 kg). The difference between body mass at the beginning and the end of therapy ranged from 0 to 21 kg (median: 1.0 kg, IQR 0–2 kg). The minimum single MSC dose per kg of body mass ranged from 0.5 × 10^6^ to 1.875 × 10^6^ (median: 0.91 × 10^6^, IQR 0.77 × 10^6^–1.0 × 10^6^). The maximum single MSC dose per kg of body mass ranged from 0.5 × 10^6^ to 2.14 × 10^6^ (median: 1.0 × 10^6^, IQR 0.87 × 10^6^–1.17 × 10^6^). The minimum total MSC dose, calculated as the minimum cell count administered in one injection divided by the maximum body mass during one treatment course, ranged from 1.2 × 10^6^ to 11.5 × 10^6^ WJ-MSCs/kg (median: 4.1 × 10^6^ WJ-MSCs/kg, IQR 2.9 × 10^6^–5.0 × 10^6^ WJ-MSCs/kg). The maximum total MSC dose, calculated by dividing the maximum cell count administered in one injection by the minimum body mass during one treatment course, ranged from 1.3 × 10^6^ to 13.6 × 10^6^ WJ-MSCs/kg (median: 4.6 × 10^6^ WJ-MSCs/kg, IQR 3.2 × 10^6^–5.9 × 10^6^ WJ-MSCs/kg).

Ninety-eight (91.6%) patients in the study population received only one course of therapy. Nine patients (8.3%) with very good response to the treatment received 1-5 additional injections in the second course.

### 3.3. Efficacy

Forty-eight of 54 patients (88.9%) included in the analysis attained some improvement in health status. Forty-eight (88.9%) patients experienced an improvement in QoL. In this subgroup of patients, the median improvement was 1.25, while in the whole population, it was 1.0 per past administration and 1.5 per collected follow-up. Twenty-one patients (38.9%) increased their self-service level. The median increase was 0.8 in this subgroup and 0 when considering the 54 patients. The number and percentage of nonresponders in subgroups categorized by the number of past WJ-MSC administrations are presented in Table 2.

### 3.4. Quality of Life

The results from the Kruskal-Wallis test indicated no difference in the average QoL per past administration and per collected follow-up between subgroups divided according to the number of past administrations. However, there was a tendency towards a difference in the average QoL per number of past administrations (*p* = 0.06). Statistical significance did not differ after binary categorization (Table 3).

### 3.5. Self-Service

According to the Kruskal-Wallis test results, there were no differences in the average self-service score per past administration and per collected follow-up between subgroups categorized by the number of past administrations. However, there was a difference in these variables after categorization by the number of collected follow-ups (Table 3). The odds ratio (OR) for lack of improvement in self-service was almost 84% lower (OR: 0.16, 95% confidence interval (CI) 0.048–0.54, *p* = 0.02) in children who received at least 4 cell infusions compared to children who received 1–3 infusions before assessment.

### 3.6. Predictive Factors for Treatment Response

There was no correlation between the average improvement in QoL per past administration or per collected follow-up and age, minimum dose per administration, maximum dose per administration, minimum total dose, or maximum total dose. There was also no difference detected in the chi-square test (Tables 4–7). In contrast, developmental breakthrough was associated with the maximum single dose, as well as with the minimum and maximum total doses. The clinical response was better in children who received at least 2.89 × 10^6^ stem cells per kilogram of body mass (Tables 5–7).

Early treatment response was correlated with average QoL improvement per following administration in both the Kendall (0.32) and Spearman methods (0.40) and the chi-square test as well (Table 8).

Out of the ten patients who did not show any improvement after the first administration (early response = 0), five did not continue the treatment, three received two additional injections, four received four additional injections (and completed course one), and two completed course one and received three or five injections in the second course. Six of these patients did not improve their QoL, and eight did not improve their self-service level. In the subgroup of patients who experienced improvement in QoL, it ranged from 0.75 to 1.75 per past administration and from 1.0 to 1.75 per collected follow-up. In two patients who experienced an improvement in their self-service level, the average self-service improvement per administration was 0.8 and 1.0, but the maximum developmental breakthrough was 3.

Out of 13 children who had an early response < 2 and received at least five infusions, 12 (92.3%) had a later response < 2. Developmental breakthrough was observed in three children from this subgroup. Two other children improved their SS by 1 point.

The average improvement in QoL per collected follow-up was better in children without epilepsy (*p* = 0.049 in Mann-Whitney *U* test), but this difference was not clinically relevant.

### 3.7. Safety

In the neurological assessment, no participant experienced adverse reactions either during the infusion or in a short time after this procedure. However, four patients' parents reported adverse events (AE) in the following days, with unknown relation to the administration of WJ-MSCs (Table 9). In three patients, the AEs were mild and temporary and passed without intervention. One AE, an increase in epileptic seizures, lead to treatment discontinuation.

AEs were not clearly associated with the cell dose. Patient #510 received a maximum WJ-MSC dose in a single administration of 0.9 × 10^6^ (<quartile 2) and a maximum WJ-MSC dose in the whole therapy of 4.55 × 10^6^ (<quartile 2). Patient #560 received 1.0 × 10^6^ (=quartile 2) and 5.0 × 10^6^ (> quartile 2), patient #592 received 1.17 × 10^6^ (> quartile 3) and 7.06 × 10^6^ (>quartile 3), and patient #386 received 1.54 × 10^6^ (> quartile 3) and 7.69 × 10^6^ (> quartile 3), respectively. Patients who experienced AEs also achieved the greatest improvement in QoL per past administration and per collected follow-up. QoL scores were as follows: 2.0 and 2.67 (both >quartile 3 equal to 1.875) for patient #510, 2.8 and 2.8 for patient #592, 1.25 and 1.25 for patient #386, and 0.5 (median level) for patient #560. Regarding their improvement in self-service per past administration, two of these patients achieved a result above quartile 3 equal to 0.6 (1.4 and 0.8); two others did not experience an improvement in their self-service.

## 4. Discussion

This study was a retrospective analysis using medical documentation obtained from a therapeutic experiment. As such, there are some limitations associated with its nature, including the lack of a control group, the lack of blinding in the evaluation, and the fact that the analysis was done retrospectively. There were also violations to the visiting schedule, and the use of concomitant therapies, including physical, pedagogical, and animal therapies. Furthermore, there was no long-term evaluation of the treatment, and a simple 6-point Likert scale was used instead of more specialized neurological scales such as GMFCS, GMFM, Up&Go, CGI, PEDI-CAT, and PedsQL. Although many authors have reported statistically significant changes in these scales after cell therapy [13], others have described them as useless for monitoring the effectiveness of rehabilitation in children with CP. In her doctoral dissertation, Depczynska analyzed 25 neurological scales validated for this purpose. She concluded that none of them met the following cumulative expectations: being intended for children diagnosed with CP and aged 7-18 years; being observational; engaging the patient on an individual basis (without comparing to healthy peers or other patients); and being sensitive to minor changes. Other expectations they failed to meet included being objective, open, and available; being modern (in accordance with the International Classification of Functioning, Disability and Health), spacious, and multiband; being useful in the assessment of the effects of the therapy; and being clearly legible, easy to perform, reliable, reactive, reproducible, and communicative [14]. We observed a similar inadequacy in the scale used in our study to measure the effectiveness of stem cell therapy in children with CP. Although there was a general improvement in the patients' condition and parental satisfaction was high, improvement was slight and divided between many areas. This led to negative results despite a real increase in the QoL of the patients, which was confirmed by healthcare professionals conducting concomitant therapies. This has inspired us to seek new scales dedicated to patients with CP treated with stem cells. An indirect proof of the need for better scales is the underregistration and underreporting of stem cell clinical trials for neurological disorders [15]. A similar methodology involving the use of a 4-point scale was used by Sharma et al. [16].

Other limitations regard technological issues. The procedure currently used to identify MSCs based on the in vitro self-renewal and multipotential evaluation of CFU-F clones only sheds light on the in vitro properties of putative MSCs. The CFU-F assay is not a definitive method for proving the existence of stem cells. In fact, the isolation of MSCs according to the criteria of the International Society for Cell & Gene Therapy produces heterogeneous, nonclonal cultures of stromal cells containing stem cells with different multipotential properties, committed progenitors, and differentiated cells [17–19]. The procedure described by Dominici et al. that we have used for 13 years in our laboratory is only one of the many options available. To the best of our knowledge, a definitive method has not yet been established, and no alternative is widely accepted. Therefore, the term “mesenchymal stem cells” used in this paper should rather be interpreted as “stem cells displaying mesenchymal features.”

Another limitation of the present study was the need to calculate an approximate stem cell dose, caused by the fluctuation in body mass observed in the patients. It was also partly caused by technical and financial considerations, since every SC administration was funded by the patients' parents, and the cost was dose-dependent. Despite methodological constraints that limit the possibility of drawing categorical conclusions, our preliminary results are valuable for creating new assessment tools dedicated to stem cell therapies. At the top of the hierarchy of evidence-based medicine, and providing the best data, are meta-analyses of controlled and randomized clinical trials. Nonetheless, the results that can be obtained from real-life settings, such as those in this therapeutic experiment, have the advantage of being more similar to daily clinical practice. Furthermore, the inclusion in the present analysis of the spontaneous reports delivered by the patients' parents revealed that the areas in which the patients improved are related to the clinical manifestation of the disease [20].

Another limitation of this study is the high number of missed follow-ups. We were not able to verify if follow-ups were skipped selectively, and therefore, we were not able to assess bias at this stage. The characteristics of the children included in the quantitative analysis were similar to those of the general population. However, even if we assumed that the health status of excluded children did not improve, our results are still encouraging. Some effectiveness was indicated for 48 of 92 (52.2%) patients who had two administrations and therefore at least one expected follow-up.

In general, our results are similar to those obtained in clinical trials. Chen et al. observed an improvement in short-term motor functions in two studies involving a total of 111 children with CP. The patients were treated with allogeneic stem cells obtained from the olfactory bulb of aborted human foetuses, as well as autologous bone marrow MSCs. The cells were cultured and propagated in vitro and differentiated into neural stem cells [21, 22]. In the first study, the authors observed an improvement in the Gross Motor Function Measure-66 (GMFM-66) that was significantly better than the result obtained using only rehabilitation. However, there was no difference in care measured on the Caregiver Questionnaire Scale. In the second study, the authors noted an improvement in motor function on the GMFM-66, with no difference in language on the Gesell Language Developmental Quotient (Gesell LDQ). In 2012, Luan et al. observed an improvement in motor functions on the GMFM-66 and the Fine Motor Scale of the Peabody Developmental Motor Scales (PDMS-FM). The authors also observed an improvement in cognition according to an investigator-developed nonvalidated checklist [23]. In 2013, Min et al. assessed the efficacy and safety of allogenic UCB administration in children with CP. This study compared 3 parallel arms: UCB+erythropoietin, placebo+erythropoietin, and double placebo [24]. Patients in the UCB group had statistically significantly higher scores on the Gross Motor Performance Measure (GMPM) and the mental and motor scales of the Bayley Scales of Infant Development (BSID-II) 6 months after therapy compared to the erythropoietin and placebo groups. The incidence of serious AEs did not differ between groups. However, the clinical improvement was slight. After 6 months, patients in the UCB group obtained 4.9 points more in the GMPM scale than patients in the control group. The differences in the GMFM and the BSID-II Motor Scale were not significant. Differences were also noted in the Functional Independence Measure (WeeFIM) Social Cognition Scale and the BSID-II Mental Scale (0.9 point and 7.7 points, respectively). Clinical results confirmed by neural imaging (F-FDG-PET/CT) revealed differential activation and deactivation patterns between the groups. In 2015, Miao et al. investigated the intrathecal administration of umbilical cord mesenchymal stem cells (UC-MSCs) in many neurological disorders, including cerebral palsy [25]. One year after the treatment, functional indices improved in 47 patients (47%): 12 patients with spinal cord injury, 11 patients with cerebral palsy, 9 patients with posttraumatic brain syndrome, 9 patients with postbrain infarction syndrome, 3 patients with spinocerebellar ataxias, and 3 patients with motor neuron disease. Side effects included headache, low-grade fever, low back pain, and lower limb pain. They were observed in 22 (22%) patients, who were treated with symptomatic therapy within 48 hours. The authors concluded that the intrathecal administration of UC-MSCs was a safe and effective way to treat neurological disorders. Also in 2015, Kang et al. evaluated the efficacy of a single intravenous dose of allogeneic UCB obtained from a cord blood bank. They observed motor improvement on the GMFM-66, as well as an increase in muscle strength on manual muscle testing [26].

In our study, the most frequently observed improvements were psychological. Half of the children with CP have an intellectual disability, which elevates their risk of premature death [27]. Thus, interventions that improve these skills are valuable, even if they do not improve physical conditions. Romanov et al. noted significant improvements in neurological status and cognitive functions after allogeneic AB0/Rh-identical UCB cell administration [28]. In the same study, 14 children (66.7%) achieved better psychological results (details unknown). These authors also noted a reduction in the frequency and severity of paroxysms in children with concomitant epilepsy, a result we observed in our study as well. In the study by Romanov et al., epileptic seizures were eliminated in one child, and in three of three patients with epilepsy, the manifestations of the disease were eradicated. In a study published in 2018, Huang et al. noticed a dose-dependent improvement in the GMFM-88 and comprehensive function assessment (CFA) with no serious AEs in 27 patients who received human UCB-MSCs [29]. In contrast, Sun et al. did not observe a significant difference in the GMFM-66, although a dosing effect was identified [30].

The partial efficacy observed in our study may be surprisingly positive in some cases, due to synergy between different methods of therapy. For example, one of the patients experienced dislocation of the hip joint caused by involuntary movements and abnormal muscle tension, which voided the effect of surgical treatment. After the 5^th^ administration of MSCs, a reduction in both was observed by the SI, the patient's mother, and the patient's paediatrician, who endorsed the proposal for the second treatment course before the bioethics committee. Although the therapy did not cure the disability, the next course gives hope for the possibility of effective surgery to prevent scoliosis.

In our study, we did not notice serious AEs. Novak et al. conducted a meta-analysis of 4 randomized and 1 nonrandomized clinical trials evaluating different stem cell therapies in a total of 328 children and young adults (<32 years) with CP. They found one death (cause unknown) and 3 other serious AEs in the stem cell group, compared to no deaths and 6 serious AEs in the control group [31]. The combined AE risk was 3% in the stem cell group, which was comparable to the 2% risk in the control group. This study is larger than ours, but it also has several limitations. It was not uniform in terms of participant age, the type of cells used and the administration methods, the type and intensity of concomitant rehabilitation, and the duration of follow-up. The last two, which are related to the nature of this disease, are difficult to eliminate without introducing ethical and methodological doubts. In the retrospective study by Feng et al., which was not included in the meta-analysis described above, the most common AEs after allogeneic UCB-stem cell administration were fever (42.6%) and vomiting (21.2%). Some AEs occurred in 26 patients (55.3%) [32]. Both of these symptoms also occurred in our patients, although with a lower incidence. In Feng's patients, all AEs disappeared after symptomatic treatment. In our study, no medical interventions were required.

In our study, the only serious AE leading to treatment discontinuation was epilepsy deterioration. Similar AEs were reported by Zali et al. in one patient after bone marrow-MSC administration [33].

The mechanism of action of MSCs in CP remains unknown. Although they are characterized by a high proliferative activity with confirmed in vitro differentiation into osteoblasts, chondroblasts, and adipocytes [34], their differentiation into neurons is controversial. Several studies have reported the possibility of differentiating human UCB-MSCs into neural cells in vitro [35, 36]. However, the main problem is the blood-brain barrier [37], which makes the homing theory less probable. The other potential mechanism involves anti-inflammatory and trophic actions [38–40].

Because of the substantial costs of therapy, ethical considerations require the identification of the prognostic factors of success related to the patient. The results from our study suggest that one of them may be epilepsy, which is an expression of severe clinical condition. On the other hand, in 5 out of 20 children with epilepsy, the number of seizures decreased, suggesting that the therapy may be beneficial regardless of improvement in QoL and SS. The second factor that should be analyzed in the future is the type of cerebral palsy. This analysis was impossible for us since the type of disease was frequently missing in the medical documentation available for our study. The third factor worth considering is genetics. In 2015, Wang et al. compared the effect of UC-MSC on the motor functions of identical twins with CP [41]. Eight pairs of homozygous twins with CP were assessed with the GMFM and FMFM scales before and 6 months after allogenic UC-MSC administration. All of them significantly improved in the GMFM scale, but not in the FMFM scale. The improvements in motor functions between two individuals of an identical pair but not among twin pairs were correlated. This result suggests that genetic factors contribute to the efficacy of UC-MSC administration in children with CP and may explain the interpersonal differences observed in our study. Another important issue that should be evaluated in the future is the timing of the stem cell administration, since animal studies suggest that early interventions are the most successful [42]. The future perspective includes also administration of allogeneic blood obtained from sibling, MSC-derived exosomes, and allogeneic cord blood. We are considering also a small clinical trial in the current model.

The intravenous administration of WJ-MSCs seems to be a safe and effective procedure that improves gross motor functions, muscle tension, communication, attention, and cognitive functions in children with CP. In our study, this led to an improvement in the QoL of the children according to the parents, third-party therapists, and the SI. However, the clinical response to the treatment varied from patient to patient. In some children, the therapeutic response was remarkable. This, in view of the severe effects of the disease and the poor therapeutic options, makes MSC administration a promising alternative. Nonetheless, further studies using more specific scales are required.

The results from our study suggest several practically important conclusions:

- Even if several follow-ups indicate no improvement, developmental breakthrough is still possible
- Low clinical improvement after the first administration may predict low effectiveness of further therapy, although it does not preclude developmental breakthrough between two consecutive assessments. This suggests that parents should be informed after the first assessment about the limited perspectives for success with further treatment
- The next crucial checkpoint is the assessment after the 3^rd^ administration. A poor response at this stage should discourage the continuation of therapy
- The minimal effective dose needed to observe improvement is 4 × 10^6^ MSCs/kg per treatment course. Clinical effects may not be noticeable before this total dose is reached

## Figures and Tables

**Table 1:** Clinical (SI) and parental qualitative assessment of improvement in different areas.

Area	Clinical assessment		Parental assessment	
	Improvement: *n* (%) *N* = 54	*p* value	*n* (%) *N* = 23	*p* value
Muscle tension	26 (48.1)	0.000001	12 (52.2)	0.0015
Muscle strength	8 (14.8)	0.007	4 (17.4)	NS
Gross motor development	9 (16.7)	0.004	11 (47.8)	0.0026
Fine motor development	4 (7.4)	NS (0.07)	5 (21.7)	NS (0.07)
Nutritional functions	4 (7.4)	NS (0.07)	3 (13.0)	NS
Senses	4 (7.4)	NS	3 (13.0)	NS
Micturition/defecation control	2 (3.7)	NS	1 (4.3)	NS
Sleeping	1 (1.9)	NS	4 (17.4)	NS
Circulation	2 (3.7)	NS	1 (4.3)	NS
Epilepsy attacks^∗^	4^∗^ (12.9)	NS	3^∗^ (9.7)	NS
Drug dosage	1 (1.9)	NS	4 (17.4)	NS
Emotions	1 (1.9)	NS	9 (39.1)	0.008
Communication	12 (22.2)	0.0009	13 (56.5)	0.0009
Attention	10 (18.5)	0.004	10 (43.5)	0.004
Cognitive functions	33 (61.1)	<0.000001	10 (43.5)	0.004
Engagement (motivation/initiative/cooperation)	3 (5.6)	NS	12 (52.2)	0.0015
Social interactions	5 (9.2)	0.04	12 (52.2)	0.0015
Number of areas	17	9 significant +2 with tendency	17	8 significant +1 with tendency

^∗^Subgroup with epilepsy; *p* value is given for sign test. NS: not significant; deteriorations are reported as adverse events.

**Table 2:** Number and percentage of nonresponders in subgroups categorized by number of past WJ-MSC administrations.

	Number of past WJ-MSC administrations								
	Course 1					Course 2			
	1	2	3	4	5	6	7	8	9
Number of patients	9	5	4	29	2	0	2	1	2
QoL nonresponders	2 (22.2%)	1 (20.0%)	2 (50%)	2 (6.9%)	0 (0%)	0	0	0	0
Self-service nonresponders	8 (88.9%)	4 (80.0%)	4 (100%)	16 (55.2%)	1 (50%)	0	0	0	0
Total nonresponders	2 (22.2%)	1 (20.0%)	2 (50%)	2 (6.9%)	0 (0%)	0	0	0	0

QoL: quality of life; WJ-MSC: Wharton's jelly mesenchymal stem cells.

**Table 3:** *p* value for Mann-Whitney *U* test comparing average quality of life in subgroups categorized by number of past WJ-MSC administrations and collected follow-ups.

		Number of past administrations					Number of collected follow-ups			
		1 vs. >1	≤2 vs. >2	≤3 vs. >3	≤4 vs. >4	≤5 vs. >5	1 vs. >1	≤2 vs. >2	≤3 vs. >3	≤4 vs. ≥5
		*n* = 9 vs. 45	*n* = 14 vs. 40	*n* = 18 vs. 36	*n* = 47 vs. 7	*n* = 49 vs. 5	*n* = 14 vs. 40	*n* = 23 vs. 31	*n* = 32 vs. 22	*n* = 51 vs. 3
QoL	PA	NS	NS	NS	NS	NS	NS	**0.01**	**0.008**	NS
	PF	NS	NS	NS	NS	NS	NS	**0.04**	**0.046**	NS
SS	PA	NS	**0.009**	**0.04**	NS	NS	**0.009**	**0.02**	**0.004**	NS
	PF	NS	**0.007**	**0.04**	NS	NS	**0.007**	**NS (0.053)**	**0.01**	NS

QoL: average improvement in quality of life; SS: average improvement in self-service; PA: per past administration; PF: per collected follow-up.

**Table 4:** Efficacy of treatment in subgroups identified on the basis of minimal cell dose per administration.

Minimal dose (×10^6/^/kg)	*N*	Average QoL response per one administration (*p* = not significant)					Developmental breakthrough^∗^ (*p* = not significant)						Proportion of patients with developmental breakthrough
		<1	1 ≤ *n* < 2	2 ≤ *n* < 3	3 ≤ *n* < 4	≥4	0	1	2	3	4	5	
≤0.77 (≤Q1)	14	**6**	**5**	**2**	1	0	**8**	**4**	**1**	0	1	0	7.1%
>0.77-≤0.91 (Q1-Q2)	16	**7**	**2**	**4**	3	0	**0**	**0**	**0**	3	2	0	31.25%
>0.91-≤1.0 (Q2-Q3)	12	6	4	1	**0**	**1**	9	1	1	**1**	**0**	**0**	8.3%
>1.0 (>Q3)	12	4	3	5	**0**	**0**	5	0	1	**3**	**3**	**0**	50%

QoL: quality of life; Q: quartile. ^∗^The highest improvement in quality of life between two consecutive assessments. Cells marked in bold represent categorization used in Yates' chi-square test.

**Table 5:** Treatment efficacy in subgroups identified on the basis of maximum cell dose per administration.

Maximum dose (×10^6^/kg)	*N*	Average QoL response per one administration (*p* chi^2^ = not significant)					Developmental breakthrough^∗^ (*p* chi^2^ with Yates correction = 0.03)						Proportion of patients with developmental breakthrough
		<1	1 ≤ *n* < 2	2 ≤ *n* < 3	3 ≤ *n* < 4	≥4	0	1	2	3	4	5	
≤0.87 (≤Q1)	14	**5**	**4**	**3**	1	0	**7**	**4**	**1**	1	1	0	14.3%
0.87-≤1.0 (Q1-Q2)	14	**8**	**3**	**2**	1	0	**11**	**1**	**1**	0	1	0	7.1%
1.0-≤1.17 (Q2-Q3)	14	6	4	2	**1**	**1**	9	0	1	**1**	**3**	**0**	28.6%
>1.17 (>Q3)	12	4	3	5	**0**	**0**	6	0	0	**5**	**1**	**0**	50%

QoL: quality of life; Q: quartile. ^∗^The highest improvement in quality of life between two consecutive assessments. Cells marked in bold represent categorization used in Yates' chi-square test.

**Table 6:** Treatment efficacy in subgroups identified on the basis of minimum total cell dose per kilogram of body mass during the whole treatment course.

Minimum total dose (×10^6^/kg)	*N*	Average QoL response per administration after following administrations (*p* chi^2^ with Yates correction = not significant)					Developmental breakthrough^∗^ (*p* chi^2^ with Yates correction = 0.0015)						Proportion of patients with developmental breakthrough
		<1	1 ≤ *n* < 2	2 ≤ *n* < 3	3 ≤ *n* < 4	≥4	0	1	2	3	4	5	
≤2.89 (<Q1)	14	**4**	**5**	**4**	0	1	**12**	**2**	**0**	0	0	0	0%
2.89-≤4.07 (Q1-Q2)	13	**6**	**3**	**2**	2	0	**9**	**1**	**2**	0	1	0	7.7%
4.07-≤5.0 (Q2-Q3)	16	8	3	3	**2**	**0**	8	1	0	**5**	**2**	**0**	43.8%
>5.0(>Q3)	11	5	3	3	**0**	**0**	4	1	1	**2**	**3**	**0**	45.5%

QoL: quality of life; Q: quartile. ^∗^The highest improvement in quality of life between two consecutive assessments. Cells marked in bold represent categorization used in Yates' chi-square test.

**Table 7:** Treatment efficacy in subgroups identified on the basis of maximum total cell dose per kilogram of body mass during the whole treatment course.

Maximum total dose (×10^6^/kg)	*N*	Average QoL response per one administration after following administrations (*p* chi^2^ with Yates correction = not significant)					Developmental breakthrough^∗^ (*p* chi^2^ with Yates correction = 0.01)						Proportion of patients with developmental breakthrough
		<1	1 ≤ *n* < 2	2 ≤ *n* < 3	3 ≤ *n* < 4	≥4	0	1	2	3	4	5	
≤3.23 (<Q1)	14	**4**	**5**	**4**	0	1	**12**	**2**	**0**	0	0	0	0%
3.23-≤4.65 (Q1-Q2)	13	**7**	**2**	**2**	2	0	**7**	**2**	**2**	1	1	0	15.4%
4.65-≤5.93 (Q2-Q3)	13	7	3	1	**2**	**0**	8	0	1	**1**	**3**	**0**	30.8%
>5.93 (>Q3)	14	1	7	6	**0**	**0**	6	1	0	**5**	**2**	**0**	50%

QoL: quality of life; Q: quartile. ^∗^The highest improvement in quality of life between two consecutive assessments. Cells marked in bold represent categorization used to Yates' chi-square test.

**Table 8:** Treatment efficacy in subgroups identified on the basis of improvement in quality of life after the first WJ-MSC administration.

Early response^∗^	*N* = 54		Average QoL response per one administration in whole later therapy (*p* chi^2^ with Yates correction = 0.014)					Developmental breakthrough^∗∗^ (*p* chi^2^ = 0.017)						Proportion of patients with developmental breakthrough (improvement in QoL ≥3 between two consecutive assessments)
		Discontinuation after 1^st^ assessment (*n*)	<1	1 ≤ *n* < 2	2 ≤ *n* < 3	3 ≤ *n* < 4	≥4	0	1	2	3	4	5	
0	10	1	**7**	**1**	**1**	0	0	**8**	**0**	**0**	2	0	0	20%
1	12	3	**7**	**1**	**1**	0	0	**8**	**3**	**0**	1	0	0	8.3%
2	20	4	**6**	**5**	**5**	0	0	**13**	**1**	**2**	2	2	0	20%
3	7	0	4	0	2	**1**	**0**	1	1	1	**2**	**2**	**0**	57.1%
4	3	1	0	0	0	**2**	**0**	2	0	0	**0**	**1**	**0**	33.3%
5	2	0	0	1	1	**0**	**0**	1	0	0	**0**	**1**	**0**	50%

^∗^Improvement in quality of life (QoL) after first administration. ^∗∗^The highest improvement in QoL between two consecutive assessments. Cells marked in bold represent categorization used in Yates' chi-square test.

**Table 9:** Adverse events reported by the patients' parents.

Adverse event	*n* (%)/*N* patients^∗^ *N* = 92	*n* (%)/*N* returned parental feedbacks *N* = 23
Dizziness	0	1 (4.3)
Drug dosage increased	1 (1.1)	0
Epileptic seizures more frequent (leading to discontinuation)	1 (1.1)	0
Emotional hypersensitivity	1 (1.1)	0
Engagement decreased	1 (1.1)	0
Fever	1 (1.1)	2 (8.7)
Headache	1 (1.1)	1 (1.1)
Hypersensitivity		
Emotional	0	5 (21.7)
Sensual	0	2 (8.7)
Infections	0	2 (8.7)
Muscle tonus decreased	0	
Skeletal		2 (8.7)
Smooth		1 (4.3)
Nausea and/or vomiting	2 (2.2)	2 (8.7)
Somnolence	0	1 (4.3)

^∗^ Number of patients with AE divided by number of children with at least one available follow-up.

## Data Availability

The datasets generated and/or analyzed during the current study are not publicly available due to the General Data Protection Regulation requirements but are available from the corresponding author on request, after anonymisation.
